# Assessing the impact of Delta and Omicron in German intensive care units: a retrospective, nationwide multistate analysis

**DOI:** 10.1186/s12913-024-11493-z

**Published:** 2024-09-23

**Authors:** Matthäus Lottes, Marlon Grodd, Linus Grabenhenrich, Martin Wolkewitz

**Affiliations:** 1 Department of Infectious Disease Epidemiology, Robert Koch Institute, Seestraße 10, 13353 Berlin, Germany; 2 Institute of Medical Biometry and Statistics, Medical Center-University of Freiburg, Stefan-Meier-Straße 26, 79104 Freiburg, Germany; 3 Department of Method Development, Research Infrastructure and Information Technology, Robert Koch Institute, Nordufer 20, 13353 Berlin, Germany

**Keywords:** Intensive care unit, COVID-19, Variant of concern, Multistate model, Competing risk

## Abstract

**Background:**

The spread of several severe acute respiratory syndrome coronavirus type 2 (SARS-CoV-2) variants of concern (VOCs) has repeatedly led to increasing numbers of coronavirus disease 2019 (COVID-19) patients in German intensive care units (ICUs), resulting in capacity shortages and even transfers of COVID-19 intensive care patients between federal states in late 2021. In this respect, there is scarce evidence on the impact of predominant VOCs in German ICUs at the population level.

**Methods:**

A retrospective cohort study was conducted from July 01, 2021, to May 31, 2022, using daily nationwide inpatient billing data from German hospitals on COVID-19 intensive care patients and SARS-CoV-2 sequence data from Germany. A multivariable Poisson regression analysis was performed to estimate the incidence rate ratios (IRRs) of transfer (to another hospital during inpatient care), discharge (alive) and death of COVID-19 intensive care patients associated with Delta or Omicron, adjusted for age group and sex. In addition, a multistate approach was used for the clinical trajectories of COVID-19 intensive care patients to estimate their competing risk of transfer, discharge or death associated with Delta or Omicron, specifically concerning patient age.

**Results:**

A total of 6046 transfers, 33256 discharges, and 12114 deaths were included. Poisson regression analysis comparing Omicron versus Delta yielded an estimated adjusted IRR of 1.23 (95% CI 1.16–1.30) for transfers, 2.27 (95% CI 2.20–2.34) for discharges and 0.98 (95% CI 0.94–1.02) for deaths. For ICU deaths in particular, the estimated adjusted IRR increased from 0.14 (95% CI 0.08–0.22) for the 0–9 age group to 4.09 (95% CI 3.74–4.47) for those aged 90 and older compared to the reference group of 60-69-year-olds. Multistate analysis revealed that Omicron was associated with a higher estimated risk of discharge for COVID-19 intensive care patients across all ages, while Delta infection was associated with a higher estimated risk of transfer and death.

**Conclusions:**

Retrospective, nationwide comparisons of transfers, discharges and deaths of COVID-19 intensive care patients during Delta- and Omicron-dominated periods in Germany suggested overall less severe clinical trajectories associated with Omicron. Age was confirmed to be an important determinant of fatal clinical outcomes in COVID-19 intensive care patients, necessitating close therapeutic care for elderly people and appropriate public health control measures.

## Background

The coronavirus disease 2019 (COVID-19) pandemic has posed major challenges to the German health care system and its health care professionals [1–4]. In particular, intensive care units (ICUs) have been severely affected during periods of increasing case numbers, with total daily occupancy even surpassing 5000 adult COVID-19 patients distributed across approximately 1300 adult ICUs nationwide [5]. With respect to the severity of the clinical condition of COVID-19 intensive care patients, the daily nationwide proportion of those requiring invasive ventilation or extracorporeal membrane oxygenation ranged mostly between 50% and 70% until early 2022 and then remained below 40% with few exceptions [6]. Consequently, efficient resource allocation and even interhospital transfers of COVID-19 intensive care patients between federal states have been crucial to avoid capacity shortages in German ICUs [7–9].

One of the main factors leading to periods of critical capacity shortage in ICUs was the spread of several severe acute respiratory syndrome coronavirus type 2 (SARS-CoV-2) variants of concern (VOCs) in the German population [10, 11]. For example, by the end of 2020, the Delta VOC was associated with an increased risk of hospitalization and fatal clinical outcomes compared with the previously dominant Alpha VOC [12–14]. ICU occupancy and ventilatory support also subsequently increased [6, 15]. In contrast, the Omicron VOC, first reported in South Africa and rapidly circulating in Germany in late 2021, led to reduced disease severity, but its sublineages demonstrated high transmissibility and immune escape [16–20]. As a result, the number of new infections in the German population has risen sharply, as has the number of COVID-19 intensive care patients, which has even necessitated their nationwide allocation [21, 22]. In this respect, a study on the impact of predominant VOCs in German ICUs from a national perspective has not yet been conducted.

Therefore, our primary objective was to retrospectively assess the impact of Delta and Omicron in German ICUs at the population level. To this end, we focused on transfer (to another hospital during inpatient care), discharge (alive) and death as potential endpoints of an ICU stay for COVID-19 patients. Consequently, we first aimed to estimate the transfer, discharge and death rates of these patients during the periods in which Delta and Omicron dominated the pandemic in Germany. Furthermore, we also aimed to estimate the corresponding risk of transfer, discharge and death by using the aforementioned rates to model the clinical trajectories of COVID-19 patients in German ICUs during the predominance of Delta and Omicron [23, 24].

## Methods

### Study design and population

We conducted a retrospective cohort study based on inpatient billing data from the Institute for the Hospital Remuneration System GmbH (InEK). These data are regularly submitted to the InEK by all German hospitals in accordance with the Hospital Remuneration Act; thus, we consider representative full coverage of all inpatient cases [25]. However, it was not possible to provide us with individual patient-level information, so we obtained the daily nationwide number of prevalent (treated in the ICU), transferred (to another hospital during inpatient care), discharged (alive) and dead COVID-19 intensive care patients (laboratory confirmed diagnosis according to the International Statistical Classification of Diseases and Related Health Problems, 10th revision, German Modification (ICD-10-GM) code U07.1!) by age group (0–9, 10–19, …, 90+) and sex (female or male) from July 1, 2021, to May 31, 2022. As the InEK only provides data on completed inpatient treatments, underreporting of daily numbers is to be expected at the end of this observation period, which was therefore limited to April 30, 2022. We further restricted our study to calendar periods when Delta (September 13, 2021 (beginning of calendar week 37) to November 21, 2021 (end of calendar week 46)) and Omicron (February 14, 2022 (beginning of calendar week 7) to April 30, 2022) were predominant, based on SARS-CoV-2 sequence data from Germany [11]. These calendar periods served as surrogate measures for the effects of Delta and Omicron in our analysis and are hereafter denoted as VOC dominance. Accordingly, we presumed that all COVID-19 intensive care patients were infected with Delta or Omicron during the respective calendar period, although this could not be verified based on InEK data. Our analysis included a total of 6046 transfers (Delta: 2209, Omicron: 3837), 33256 discharges (Delta: 7570, Omicron: 25686), and 12114 deaths (Delta: 4313, Omicron: 7801).

### Statistical analysis

We first performed a multivariable Poisson regression analysis to estimate the adjusted incidence rate ratios (IRRs) of transfer, discharge, and death in COVID-19 intensive care patients associated with VOC dominance. In doing so, we set an offset for the population size, i.e., the daily (prevalent) number of COVID-19 intensive care patients during the selected calendar periods, and fitted a separate model for the effect of VOC dominance on each outcome studied, adjusted for age group and sex based on InEK data (Table 1). Delta served as the reference for VOC dominance, as it was prior to Omicron. The 60–69 age group was chosen as the age reference, as this group had the highest number of prevalent COVID-19 intensive care patients. Female COVID-19 intensive care patients served as the sex reference but for no specific reason. For interpretation, an estimated adjusted IRR above 1 (below 1) indicates a higher (lower) event rate of transfer, discharge and death in the available categories of VOC dominance, age group and sex compared to their reference.

**Table 1:** Incidence rate ratio, 95% confidence interval and *p*-value for transfer (to another hospital during inpatient care), discharge (alive) and death of COVID-19 intensive care patients in Germany by predominant SARS-CoV-2 variant of concern, age group and sex

Predictors	Transfer			Discharge			Death		
	Incidence Rate Ratio	95% CI ^a^	*p* ^ b^	Incidence Rate Ratio	95% CI ^a^	*p * ^b^	Incidence Rate Ratio	95% CI ^a^	*p * ^b^
Delta	1.00	*Reference*		1.00	*Reference*		1.00	*Reference*	
Omicron	1.23	1.16–1.30	< 0.001	2.27	2.20–2.34	< 0.001	0.98	0.94–1.02	0.298
Age 0–9	0.57	0.41–0.76	< 0.001	1.88	1.72–2.05	< 0.001	0.14	0.08–0.22	< 0.001
Age 10–19	1.54	1.17–1.99	0.001	2.40	2.15–2.67	< 0.001	0.34	0.21–0.51	< 0.001
Age 20–29	1.54	1.27–1.84	< 0.001	2.33	2.15–2.53	< 0.001	0.30	0.22–0.41	< 0.001
Age 30–39	1.52	1.33–1.72	< 0.001	1.76	1.65–1.88	< 0.001	0.41	0.34–0.49	< 0.001
Age 40–49	1.35	1.21–1.50	< 0.001	1.40	1.32–1.48	< 0.001	0.52	0.46–0.58	< 0.001
Age 50–59	1.19	1.09–1.30	< 0.001	1.17	1.12–1.23	< 0.001	0.70	0.65–0.76	< 0.001
Age 60–69	1.00	*Reference*		1.00	*Reference*		1.00	*Reference*	
Age 70–79	0.90	0.83–0.98	0.015	1.02	0.98–1.06	0.319	1.50	1.42–1.59	< 0.001
Age 80–89	0.98	0.90–1.07	0.640	1.31	1.26–1.37	< 0.001	2.58	2.44–2.72	< 0.001
Age 90+	1.18	0.99–1.41	0.065	2.01	1.87–2.16	< 0.001	4.09	3.74–4.47	< 0.001
Female	1.00	*Reference*		1.00	*Reference*		1.00	*Reference*	
Male	0.90	0.85–0.95	< 0.001	0.83	0.81–0.85	< 0.001	1.04	1.00–1.08	0.075

a Confidence interval

b *P*-value

**Fig. 1:**
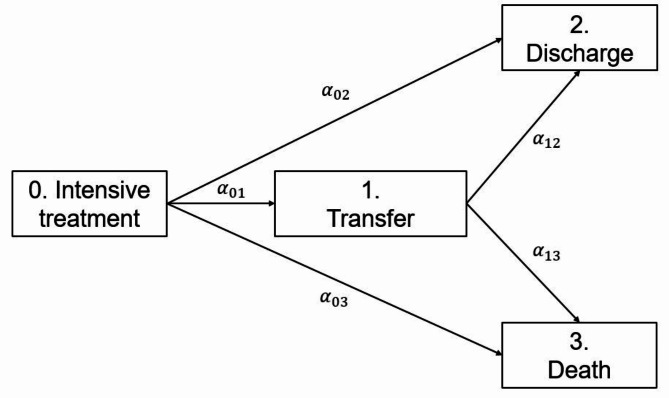
Graphical representation of a multistate model for the clinical trajectories and outcomes of COVID-19 intensive care patients in Germany based on nationwide inpatient billing data from the Institute for the Hospital Remuneration System GmbH (InEK)

Second, we used a multistate approach (Fig. 1) to model the clinical trajectories of COVID-19 patients in the ICU, accounting for the temporal dependency of the competing events of transfer, discharge and death [26, 27]. Accordingly, (prevalent) patients were receiving ICU treatment (state 0). The patients remained in this state until they were either transferred to another hospital as part of inpatient care (state 1) or discharged from the ICU (state 2) unless they died there (state 3). Based on InEK data, COVID-19 patients were also either discharged from or died in the ICU after an interhospital transfer (from state 1 to state 2 or state 3), which certainly did not apply to every patient. The multistate model depends on the hazard rates ($\:{\alpha\:}_{01},\:{\alpha\:}_{02},\:{\alpha\:}_{03},\:{\alpha\:}_{12},\:{\alpha\:}_{13}$) between the states of ICU treatment, transfer, discharge and death. The hazard rates, in turn, depend on the available categories of VOC dominance, age group and sex. As we were unable to draw any conclusions about individual patient histories, the following assumptions were made: in each of the available categories, the hazard rates remained constant over time, discharge and death rates were independent of interhospital transfers $\:{(\alpha\:}_{02}\:=\:{\alpha\:}_{12},\:{\alpha\:}_{03}\:=\:{\alpha\:}_{13})$, an interhospital transfer occurred only once during the inpatient treatment of a COVID-19 intensive care patient, and as mentioned above, discharges (state 2) and deaths (state 3) always occurred in the ICU. Based on these assumptions, we adapted the approach of von Cube et al., who exemplified how all transition probabilities of a multistate model can be explicitly calculated based on time-constant transition-specific hazard rates to provide initial insights into principle time dynamics [28]. Hence, we converted the constant hazard rates of our multistate model (Fig. 1) into cumulative probabilities, i.e., estimated risks for the effect of VOC dominance on each outcome studied, adjusted for either age group or sex depending on time (ICU day), noted as *t*﻿:

$$\:{\alpha\:}_{0}=-\left({\alpha\:}_{01}+{\alpha\:}_{02}+{\alpha\:}_{03}\right)$$

$$\:{\alpha\:}_{1}=-\left({\alpha\:}_{12}+{\alpha\:}_{13}\right)$$

$$\:{P}_{00}\left(t\right)={e}^{{\alpha\:}_{0}\cdot\:t}$$

$$\:{P}_{01}\left(t\right)=\frac{{\alpha\:}_{01}}{{\alpha\:}_{0}-{\alpha\:}_{1}}\left({e}^{{\alpha\:}_{0}\cdot\:t}-{e}^{{\alpha\:}_{1}\cdot\:t}\right)$$

$$\begin{array}{l}\:{P_{02}}\left( t \right) = \frac{{\alpha {\:_{01}}\alpha {\:_{12}}}}{{\alpha {\:_0}\alpha {\:_1}}}\left( {1 - {e^{\alpha {\:_0} \cdot \:t}}} \right) + \frac{{\alpha {\:_{01}}\alpha {\:_{12}}}}{{\alpha {\:_0}\left( {\alpha {\:_0} - \alpha {\:_1}} \right)}}\left( {{e^{\alpha {\:_0} \cdot \:t}} - {e^{\alpha {\:_1} \cdot \:t}}} \right) - \frac{{\alpha {\:_{02}}}}{{\alpha {\:_0}}}\left( {1\: - {e^{\alpha {\:_0} \cdot \:t}}} \right)\end{array}$$

$$\begin{array}{l}\:{P_{03}}\left( t \right) = \frac{{\alpha {\:_{01}}\alpha {\:_{13}}}}{{\alpha {\:_0}\alpha {\:_1}}}\left( {1 - {e^{\alpha {\:_0} \cdot \:t}}} \right) + \frac{{\alpha {\:_{01}}\alpha {\:_{13}}}}{{\alpha {\:_0}\left( {\alpha {\:_0} - \alpha {\:_1}} \right)}}\left( {{e^{\alpha {\:_0} \cdot \:t}} - {e^{\alpha {\:_1} \cdot \:t}}} \right) - \frac{{\alpha {\:_{03}}}}{{\alpha {\:_0}}}\left( {1\: - {e^{\alpha {\:_0} \cdot \:t}}} \right)\end{array}$$

Given the present scenario of competing events, the risk of being transferred to another hospital was estimated using the cumulative incidence function:

$$\:CI{F}_{01}\left(t\right)=\frac{{\alpha\:}_{01}}{{\alpha\:}_{0}}\left(1-{e}^{-{\alpha\:}_{0}\cdot\:t}\right)$$

**Fig. 2:**
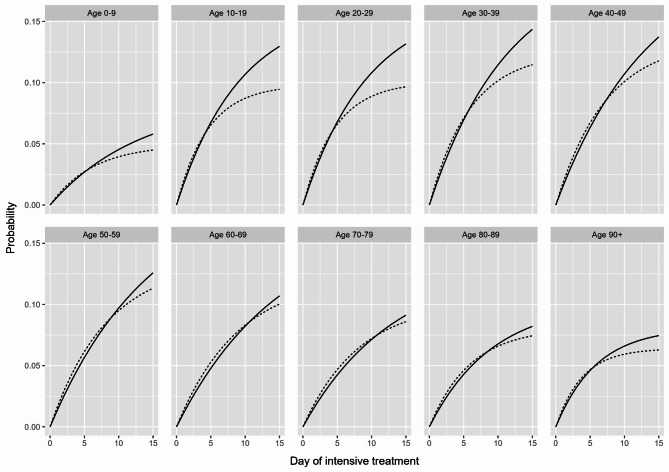
Line plot for the estimated risk of transfer (to another hospital during inpatient care) for COVID-19 intensive care patients in Germany by predominant SARS-CoV-2 variant of concern (dashed line: Omicron, solid line: Delta) and age group up to Day 15 after initial intensive treatment

**Fig. 3:**
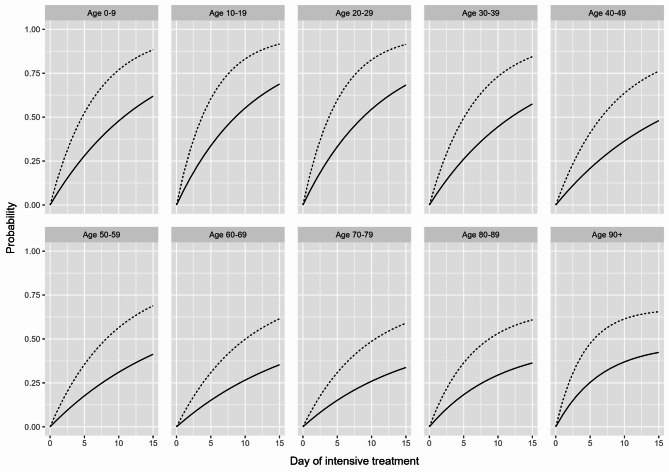
Line plot for the estimated risk of discharge (alive) for COVID-19 intensive care patients in Germany by predominant SARS-CoV-2 variant of concern (dashed line: Omicron, solid line: Delta) and age group up to Day 15 after initial intensive treatment

**Fig. 4:**
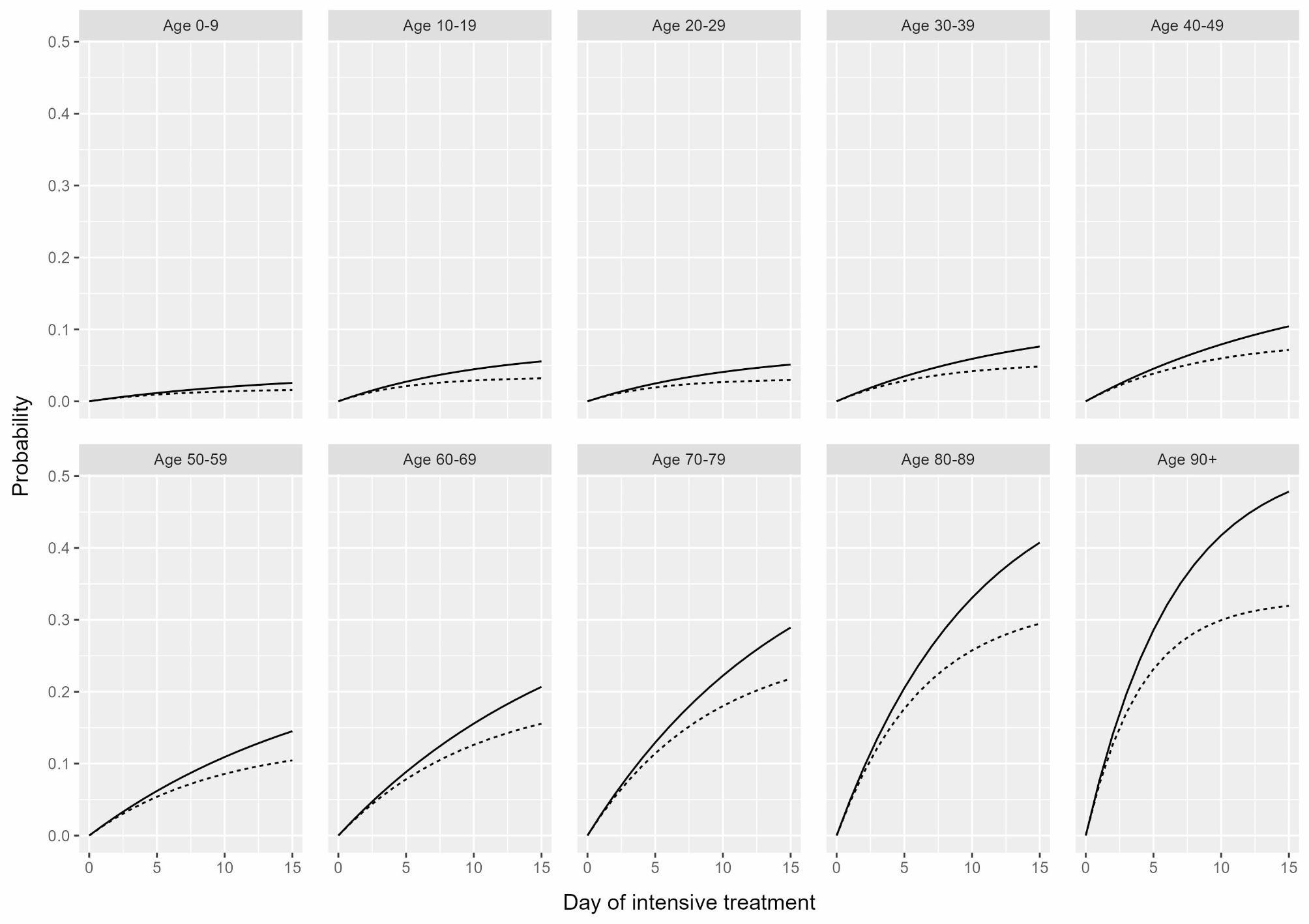
Line plot for the estimated risk of death for COVID-19 intensive care patients in Germany by predominant SARS-CoV-2 variant of concern (dashed line: Omicron, solid line: Delta) and age group up to Day 15 after initial intensive treatment

Consequently, the risk of being discharged from or dying in the ICU was also estimated using the cumulative incidence function $\:({CIF}_{02}\left(t\right)=\:{P}_{02}\left(t\right),{CIF}_{03}\:\left(t\right)={P}_{03}\left(t\right))$. For our results, we primarily considered the respective risk of VOC dominance up to Day 15 after initial ICU treatment (Figs. 2, 3 and 4), although longer stays were of course possible, and by age group, since age has been found to be a major determinant of COVID-19-related in-hospital mortality [29–31].

## Results

### IRRs of transfer, discharge and death of COVID-19 intensive care patients

As shown in Table 1, the transfer and discharge rates were comparatively higher for COVID-19 intensive care patients in the Omicron-dominated period, with estimated adjusted IRRs of 1.23 (95% CI 1.16–1.30) and 2.27 (95% CI 2.20–2.34), respectively. The death rate of COVID-19 intensive care patients was similar in the Delta- and Omicron-dominated periods, with an estimated adjusted IRR of 0.98 (95% CI 0.94–1.02).

Compared to those in the 60–69 years age group, younger COVID-19 intensive care patients predominantly had higher transfer rates, whereas the opposite was found in older age groups (Table 1). For discharge, the estimated adjusted IRR peaked at 2.40 (95% CI 2.15–2.67) in the 10–19 age group, then steadily decreased until the 60–69 age group, and then increased again for older groups. For death, younger COVID-19 intensive care patients had lower rates than did those in the 60–69 age group, whereas the opposite was found for older age groups, with an estimated adjusted IRR for the 90 + age group increasing to 4.09 (95% CI 3.74–4.47). Compared to female COVID-19 intensive care patients, male patients had lower transfer and discharge rates but a similar death rate, with an estimated adjusted IRR of 1.04 (95% CI 1.00–1.08).

### Risk of transfer, discharge and death of COVID-19 intensive care patients

As shown in Fig. 2, the estimated risk of transfer at the beginning of ICU treatment was slightly higher for COVID-19 patients during the Omicron-dominated period across all age groups. However, with longer ICU treatment, the estimated risk of transfer was higher during the Delta-dominated period and was still above 0.12 on Day 15 after the initial ICU treatment for 10- to 59-year-olds.

As shown in Fig. 3, the estimated risk of discharge for COVID-19 intensive care patients decreased with increasing age but was consistently higher across all age groups during the Omicron-dominated period. For example, the estimated risk of discharge on Day 10 after initial ICU treatment during the Omicron-dominated period was 0.77 for the 0–9 age group and 0.53 for the 80–89 age group, while during the Delta-dominated period, it was 0.48 (0–9 age group) and 0.29 (80–89 age group).

As shown in Fig. 4, the estimated risk of death for COVID-19 intensive care patients increased sharply with age but was consistently lower across all age groups during the Omicron-dominated period. For example, the estimated risk of death on Day 10 after initial ICU treatment during the Omicron-dominated period was 0.01 for the 0–9 age group and 0.26 for the 80–89 age group, while during the Delta-dominated period, it was 0.02 (0–9 age group) and 0.33 (80–89 age group).

## Discussion

In this retrospective cohort study, we aimed to assess the impact of Delta and Omicron in German ICUs on the population level using nationwide inpatient billing data provided by the InEK. Thus, we compared calendar periods when Delta and Omicron dominated the pandemic in Germany and its ICUs to estimate the associated IRRs and risks of transfer (to another hospital during inpatient care), discharge (alive) and death in COVID-19 intensive care patients. We would like to point out that these ICU endpoints are multifactorial and that despite adjustment for age group and sex, the possible effects of vaccination coverage and status, type of medical treatment, capacity of ICU health workforce, availability of medical devices and others could not be addressed. We therefore advise against interpreting our effect estimates for VOC dominance as causal unless rigorous confounding adjustment has been made [32]. However, since we presumably covered the entire cohort of COVID-19 intensive care patients during the selected calendar periods, our results may certainly be considered representative of the overall differential impact of Delta and Omicron in the German ICU setting.

Following on from this, the results of our multivariable Poisson regression analysis showed that Omicron was associated with higher transfer and discharge rates in COVID-19 intensive care patients than was Delta, while the death rates were similar during both observed calendar periods. One plausible explanation could be the comparatively better clinical condition and prognosis of the ‘Omicron patients,’ which allowed ICU capacity to be freed up more frequently than during the Delta-dominated period. On the other hand, this could also indicate overloaded ICUs and the need to free up beds in view of the sharp increase in COVID-19 admissions, which of course requires patients whose clinical condition allows them to be transferred or discharged [6, 33]. Although disease severity cannot be inferred from transfer, discharge and death rates alone, our nationwide representation may hint at overall less severe clinical trajectories when Omicron dominated the pandemic in Germany and its ICUs. With respect to age, older COVID-19 intensive care patients generally had lower transfer and discharge rates but a higher death rate than younger patients did. These findings support studies pointing toward the strong relevance of age, particularly in the context of COVID-19-related in-hospital mortality and the consequent need for close therapeutic care of elderly people [29–31].

The results of our multistate analysis further demonstrated the overall differential impact of the VOC studied, with Omicron associated with a comparatively lower estimated risk of transfer and death in COVID-19 intensive care patients, while Delta was associated with a generally lower estimated risk of ICU discharge across all age groups. Since our estimates were based on all inpatient COVID-19 cases treated in German ICUs during the observed calendar periods, they may well be seen as representative indications of rather less severe or fatal clinical courses during the Omicron-dominated period. This finding therefore appears to support previous studies based on inpatient data from 69 to 89 German Helios hospitals, respectively, reporting a substantially lower risk for ICU treatment, mechanical ventilation and in-hospital mortality during the Omicron period [34, 35]. Our results also appear to be consistent with studies based on inpatient data outside of Germany, which revealed a higher incidence of severe pneumonia in patients with Delta infection and that those with Omicron infection had a comparatively lower risk of severe disease, i.e., less invasive ventilation and lower in-hospital mortality [36–39]. Once again, the relevance of the age of COVID-19 patients for dying in the ICU was apparent, which underpins the necessity for close therapeutic care in elderly people, regardless of which VOC was spreading [29–31]. From a public health perspective, this also substantiated the fundamental need for appropriate control measures to reduce the risk of SARS-CoV-2 transmission and severe COVID-19 disease [40].

Finally, we would like to address the peculiarity of converting rates to risks in the presence of competing events, as a contradiction might be suspected when comparing our results from Poisson regression and multistate analysis. This apparent contradiction is explained by the fact that, first, rate and risk are different measures, and second, the estimated risk for each competing event depends on all corresponding hazard rates of the multistate model [41]. To clarify, Omicron was associated with a comparatively higher transfer rate in COVID-19 intensive care patients (IRR > 1.0). However, when the competing discharge event was also considered (IRR > 1.0), this transfer rate was converted to a lower estimated risk of transfer with Omicron. Likewise, while Omicron was associated with a similar death rate in COVID-19 intensive care patients as was Delta (IRR ∼ 1.0), the estimated risk of death was lower with Omicron because affected patients were discharged at a higher rate (IRR > 1.0). Thus, the modeled clinical trajectories of COVID-19 intensive care patients demonstrate the loss of one-to-one correspondence between the rate and risk, which is a distinctive feature of competing event scenarios [42].

One of the strengths of the present study is the use of a data source that represents the inpatient billing data of all German hospitals as part of the mandatory data transmission to the InEK. These data allowed us to provide a population-level insight into the German ICU setting during the dynamics of the COVID-19 pandemic, demonstrating the overall differential impact of Delta and Omicron. Moreover, the relevance of age for COVID-19-related in-hospital mortality has been further substantiated. From a methodological perspective, our multistate approach has proven to be an appropriate means of modeling patient-state transitions in the presence of competing risks using aggregated count data. To our knowledge, there are no comparable approaches addressing the clinical trajectories and outcomes of COVID-19 intensive care patients at the German national level.

A main limitation of our study is the lack of patient-level information, which led us to make assumptions that do not hold in clinical practice, most notably assuming time-constant hazards. In addition, incomplete confounding adjustment does not allow for a causal interpretation of our effect estimates. An appropriate adjustment of our multistate model and a more differentiated analysis would therefore require comprehensive individual patient-level and ICU-specific information.

## Conclusions

Retrospective, nationwide comparisons of COVID-19 intensive care patient transfers, discharges and deaths during Delta- and Omicron-dominated periods in Germany suggested overall less severe clinical trajectories with Omicron. Age was confirmed to be an important determinant of fatal clinical outcomes in COVID-19 intensive care patients, necessitating close therapeutic care for elderly people and appropriate public health control measures.

## Data Availability

The data that support the findings of this study are available from the InEK, but restrictions apply to the availability of these data, which were used under license for the current study and are not publicly available. However, the data are available from the corresponding author upon reasonable request and with permission of the Federal Ministry of Health.

## Abbreviations

COVID-19: Coronavirus disease 2019
ICD-10-GM: International Statistical Classification of Diseases and Related Health Problems, 10th revision, German Modification
ICU: Intensive care unit
InEK: Institute for the Hospital Remuneration System GmbH
IRR: Incidence rate ratio
SARS-CoV-2: Severe acute respiratory syndrome coronavirus type 2
VOC: Variant of concern
